# Usefulness of Cellular Analysis of Bronchoalveolar Lavage Fluid for Predicting the Etiology of Pneumonia in Critically Ill Patients

**DOI:** 10.1371/journal.pone.0097346

**Published:** 2014-05-13

**Authors:** Sang-Ho Choi, Sang-Bum Hong, Hyo-Lim Hong, Sung-Han Kim, Jin Won Huh, Heungsup Sung, Sang-Oh Lee, Mi-Na Kim, Jin-Yong Jeong, Chae-Man Lim, Yang Soo Kim, Jun Hee Woo, Younsuck Koh

**Affiliations:** 1 Department of Infectious diseases, Asan Medical Center, University of Ulsan College of Medicine, Seoul, Republic of Korea; 2 Department of Pulmonary and Critical Care Medicine, Asan Medical Center, University of Ulsan College of Medicine, Seoul, Republic of Korea; 3 Department of Laboratory Medicine, Asan Medical Center, University of Ulsan College of Medicine, Seoul, Republic of Korea; 4 Asan Institute for Life Sciences, Asan Medical Center, University of Ulsan College of Medicine, Seoul, Republic of Korea; University of California Riverside, United States of America

## Abstract

**Background:**

The usefulness of bronchoalveolar lavage (BAL) fluid cellular analysis in pneumonia has not been adequately evaluated. This study investigated the ability of cellular analysis of BAL fluid to differentially diagnose bacterial pneumonia from viral pneumonia in adult patients who are admitted to intensive care unit.

**Methods:**

BAL fluid cellular analysis was evaluated in 47 adult patients who underwent bronchoscopic BAL following less than 24 hours of antimicrobial agent exposure. The abilities of BAL fluid total white blood cell (WBC) counts and differential cell counts to differentiate between bacterial and viral pneumonia were evaluated using receiver operating characteristic (ROC) curve analysis.

**Results:**

Bacterial pneumonia (n = 24) and viral pneumonia (n = 23) were frequently associated with neutrophilic pleocytosis in BAL fluid. BAL fluid median total WBC count (2,815/µL vs. 300/µL, *P*<0.001) and percentage of neutrophils (80.5% vs. 54.0%, *P* = 0.02) were significantly higher in the bacterial pneumonia group than in the viral pneumonia group. In ROC curve analysis, BAL fluid total WBC count showed the best discrimination, with an area under the curve of 0.855 (95% CI, 0.750–0.960). BAL fluid total WBC count ≥510/µL had a sensitivity of 83.3%, specificity of 78.3%, positive likelihood ratio (PLR) of 3.83, and negative likelihood ratio (NLR) of 0.21. When analyzed in combination with serum procalcitonin or C-reactive protein, sensitivity was 95.8%, specificity was 95.7%, PLR was 8.63, and NLR was 0.07. BAL fluid total WBC count ≥510/µL was an independent predictor of bacterial pneumonia with an adjusted odds ratio of 13.5 in multiple logistic regression analysis.

**Conclusions:**

Cellular analysis of BAL fluid can aid early differential diagnosis of bacterial pneumonia from viral pneumonia in critically ill patients.

## Introduction

Severe pneumonia requiring intensive care unit (ICU) admission is associated with high rates of morbidity and mortality. Delays in the provision of adequate antimicrobial therapy have been reported to be associated with excess mortality [1]–[3]; therefore, rapid and accurate etiologic diagnosis of severe pneumonia is essential for successful treatment. In recent years, bronchoscopic bronchoalveolar lavage (BAL) has been established as a useful technique for collecting lower respiratory tract specimens from the alveolar level, and can thus be used to accurately define the causative organisms of pneumonia [4]–[6]. However, a conventional culture usually takes at least a few days, and microbiological yield is often compromised by prior empirical usage of antimicrobial agents. In addition, identification of viruses and atypical organisms requires a separate etiologic work-up.

Cellular analysis of BAL fluid, including total and differential cell counts and the CD4+:CD8+ T-lymphocyte ratio, is useful for the diagnosis of various interstitial lung diseases [7]–[9]. Under an appropriate clinical setting, BAL fluid analysis can provide highly suggestive or even diagnostic information for specific interstitial lung diseases in the absence of a lung biopsy [10]. However, only a few previous studies with limited patient populations [11]–[13] have evaluated the role of cellular analysis of BAL fluid in patients with suspected pneumonia. Most of these studies focused on the differential diagnosis of pneumonia from non-infectious pulmonary diseases, not on the prediction of pneumonia etiology. BAL fluid analysis can be performed within several hours. Therefore, such analysis would be useful for guiding early treatment if it could predict the etiology of pneumonia, similar to the role of cerebrospinal fluid cellular analysis, which can reliably differentiate among meningitis etiologies. Therefore, this study investigated whether analysis of the cellular profile of BAL fluid can predict the etiology of pneumonia in critically ill patients admitted to the medical ICU.

## Methods

### Study design and setting, population, and data collection

This study was based on data from a prospective observational cohort study conducted from March 2010 to May 2013. All patients admitted to the medical ICU of Asan Medical Center, a 2,700-bed tertiary care university-affiliated hospital in Seoul, Republic of Korea, with suspected severe pneumonia were prospectively identified and monitored until discharged [14]. The data collected included patient demographics, underlying diseases or conditions, illness severity scores including Acute Physiological and Chronic Health Evaluation (APACHE) II and Sequential Organ Failure Assessment (SOFA), type of pneumonia, laboratory data including microbiological tests, length of ICU stay, and outcome. The prospectively collected data were retrospectively analyzed. This study was approved by the Institutional Review Board of Asan Medical Center and the requirement for informed consent was waived because of the observational nature of the study. All patients information was anonymized and de-identified prior to analysis.

### Inclusion and exclusion criteria

Inclusion criteria were as follows: (1) patients aged ≥18 years with a clinical diagnosis of pneumonia (see below for definition), and (2) patients who underwent bronchoscopic BAL for etiologic diagnosis of pneumonia. Exclusion criteria were as follows: (1) patients in whom the pathogen was not identified, (2) patients in whom BAL fluid analysis was impossible (due to severe neutropenia or clotting of specimen) or not performed, (3) patients with a mixed infection (identification of bacteria and virus), (4) patients who were treated with antimicrobial agents for more than 24 hours before bronchoscopic BAL, (5) patients with invasive pulmonary aspergillosis, (6) patients with mycobacterial infection, and (7) patients with *Pneumocystis jirovecii* pneumonia.

### Definitions

Pneumonia was defined as the presence of an acute infiltrate on a chest radiograph and at least one of the following: fever (temperature ≥38.0°C) or hypothermia (temperature <35.0°C), cough, pleuritic chest pain, dyspnea, and altered breath sounds on auscultation [15]. Pneumonia was categorized as community-acquired pneumonia (CAP), healthcare-associated pneumonia (HCAP), or hospital-acquired pneumonia (HAP), as defined previously [16], [17].

### Bronchoscopic BAL and BAL fluid processing and analysis

Fiberoptic bronchoscopy with BAL was performed following a standardized protocol as previously described [14]. Briefly, BAL was performed by instillation of three consecutive aliquots of sterile saline solution (20–30–30 ml) into the bronchial tree at the area that was most abnormal on the chest radiography. The right middle lobe or lingual segment was chosen in patients with bilateral diffuse infiltration. BAL fluid that was first retrieved was discarded, and BAL fluid that was subsequently retrieved was collected. The total cell count was determined using a hemocytometer. The corresponding amount of BAL fluid for 10^3^ cells was centrifuged onto a microscope slide using a Thermo Shandon Cytospin (Thermo Fisher Scientific Inc., Waltham, MA, USA), at 500 rpm for 5 minutes at room temperature. The slide was air-dried and stained with Wright-Giemsa stain. Differential cell counts that included percentages of neutrophils, lymphocytes, alveolar macrophages, and eosinophils were determined.

### Microbiological Evaluation

Bacterial, fungal, and mycobacterial cultures of endotracheal aspirates and BAL fluid were performed. Respiratory viruses were tested by a multiplex reverse-transcription polymerase chain reaction (PCR) assay using a Seeplex RV15 ACE Detection kit (Seegene Inc., Seoul, Korea) and/or shell vial culture. PCR to detect *Mycoplasma pneumoniae*, *Chlamydophila pneumoniae*, and *Legionella pneumophila*, and a urinary antigen test to detect *Streptococcus pneumoniae* and *L. pneumophilia* serogroup 1 species were also performed.

### Statistical analysis

Data were expressed as mean ± standard deviation or median and 25–75% interquartile range according to data distribution. Categorical variables were compared using the chi-square test or Fisher’s exact test as appropriate. Receiver operating characteristic (ROC) curves were constructed to determine the performances of BAL fluid cellular components, serum procalcitonin concentration, and C-reactive protein concentration for predicting bacterial pneumonia. Youden’s Index (sensitivity + specificity-1) [18] was used to select the optimal cutoff points of the ROC curve. Area under the curve (AUC), sensitivity, specificity, positive likelihood ratio and negative likelihood ratio were calculated. For positive- and negative predictive values, the prevalence of bacterial pneumonia in severe pneumonia patients admitted to the medical ICU was assumed to be 35.9%, based on our previous study [14]. Multivariable logistic regression analysis was used to identify independent predictors of bacterial pneumonia. Variables with *P* values less than 0.2 in the univariate analysis were included in the multivariate analysis. The correlation between BAL fluid white blood cell (WBC) count and APACHE II score was determined by calculating Pearson’s correlation coefficient. Significance was accepted at *P* ≤ 0.05. All tests were performed using SPSS (version 18.0; SPSS, Inc.) and GraphPad Prism (version 5; GraphPad, Inc.) software.

## Results

### Study population

**Figure 1:**
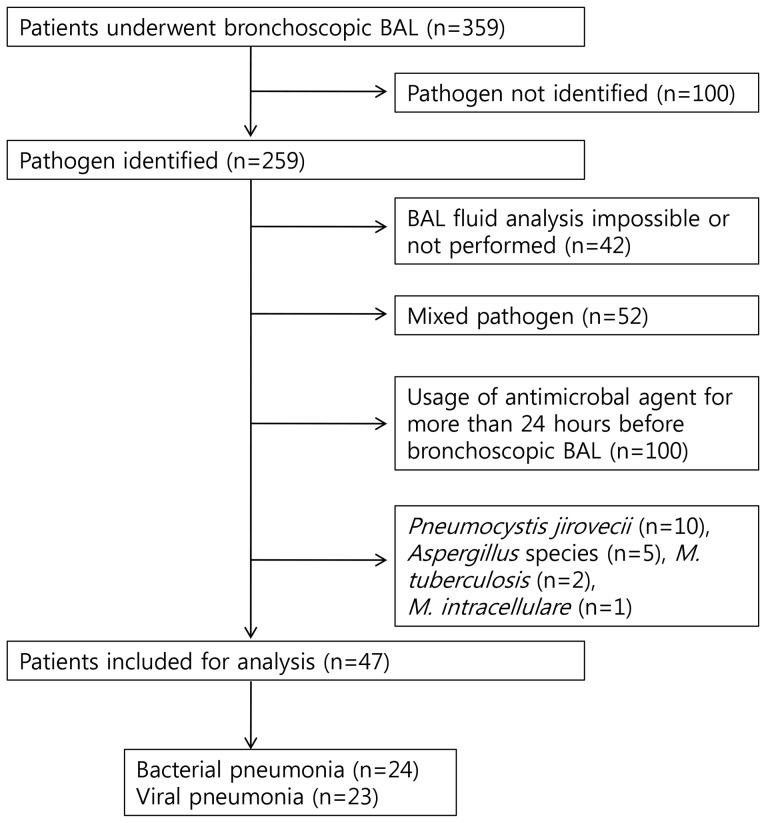
Enrollment process for patients admitted to the medical intensive care unit due to pneumonia, with reasons for exclusion.

Figure 1 shows the patient enrollment process and the reasons for exclusion. During the study period, 359 adult patients with pneumonia underwent bronchoscopic BAL (67 with CAP, 159 with HCAP, and 133 with HAP). Of these patients, 100 were excluded because the pathogen was not identified, 42 were excluded because BAL fluid analysis was not possible (due to severe neutropenia or specimen clotting) or not performed, 52 were excluded because two or more types of pathogens were identified, and 100 were excluded because they received antimicrobial therapy for more than 24 hours before bronchoscopic BAL. Ten patients with *Pneumocystis jirovecii* pneumonia, 5 patients with invasive pulmonary aspergillosis, and 3 patients with mycobacterial pneumonia were also excluded. Finally, 47 patients (24 with bacterial pneumonia and 23 with viral pneumonia) were included.

### Patient characteristics

The characteristics of the 47 patients are shown in Table 1. Thirty-two patients (68.1%) were men and the mean age was 62.1 years. Structural lung disease was the most common underlying disease (29.8%), followed by diabetes mellitus (19.0%), and hematologic malignancy/solid cancer (both 12.8%). Sixteen patients (34.0%) had CAP, 25 (53.2%) had HCAP, and 6 (12.8%) had HAP. Most baseline characteristics did not significantly differ between the bacterial pneumonia and viral pneumonia groups. By contrast, mean APACHE II (27.0±6.8 vs. 20.8±5.3, *P* = .002) and SOFA (11.3±3.7 vs. 7.6±3.2, *P* = .001) scores were significantly higher in the bacterial pneumonia group than in the viral pneumonia group. However, mortality rates, including 28-day mortality, did not significantly differ between the groups.

**Table 1:** Demographics, underlying diseases/conditions, and clinical characteristics of patients with pneumonia.

	All patients (n-47)	Bacterial pneumonia (n = 24)	Viral pneumonia (n = 23)	*P*-value
Male	32 (68.1)	18 (75.0)	14 (60.9)	0.30
Mean age ± SD, y	62.1±15.2	62.6±15.9	61.6±14.8	0.81
Underlying disease or condition^a^				
Structural lung disease	14 (29.8)	8 (33.3)	6 (26.1)	0.59
Interstitial lung disease	8 (17.0)	3 (12.5)	5 (21.7)	
COPD	4 (8.5)	3 (12.5)	1 (4.3)	
Bronchiectasis	1 (2.1)	1 (4.2)	0	
Destroyed lung due to tuberculosis	1 (2.1)	1 (4.2)	0	
Diabetes mellitus	9 (19.0)	3 (12.5)	6 (26.1)	0.29
Hematologic malignancy	6 (12.8)	2 (8.3)	4 (17.4)	0.42
Solid cancer	6 (12.8)	5 (20.8)	1 (4.3)	0.19
Stem cell transplantation	5 (10.6)	2 (8.3)	3 (13.0)	0.67
Rheumatoid arthritis				
Liver cirrhosis	2 (4.3)	2 (8.3)	0	0.49
Alcoholism	2 (4.3)	2 (8.3)	0	0.49
End-stage renal disease	2 (4.3)	2 (8.3)	0	0.49
Congestive heart failure	2 (4.3)	1 (4.2)	1 (4.3)	1.00
Solid organ transplantation	2 (4.3)	0	2 (8.7)	0.23
Cerebrovascular attack	1 (2.1)	0	1 (4.3)	0.49
Receipt of immunosuppressant	11 (23.4)	6 (25.0)	5 (21.7)	0.79
Recent chemotherapy	5 (10.6)	2 (8.3)	3 (13.0)	0.67
Active smoker	6 (12.8)	3 (12.5)	3 (13.0)	1.0
Recent surgery (within 1 month)	4 (8.5)	4 (16.7)	0	0.11
Category of pneumonia				
CAP	16 (34.0)	7 (29.2)	9 (39.1)	0.47
HCAP	25 (53.2)	12 (50.0)	13 (56.5)	0.65
HAP	6 (12.8)	5 (20.8)	1 (4.3)	0.19
APACHE II score ± SD	23.9±6.8	27.0±6.8	20.8±5.3	0.002
SOFA score ± SD	9.5±3.9	11.3±3.7	7.6±3.2	0.001
Septic shock at admission	21 (44.7)	12 (50.0)	9 (39.1)	0.45
Mechanical ventilation	40 (85.1)	22 (91.7)	18 (78.3)	0.25
Mortality				
14-day mortality	7 (14.9)	3 (12.5)	4 (17.4)	0.70
28-day mortality	13 (27.7)	8 (33.3)	5 (21.7)	0.37
60-day mortality	19 (40.4)	8 (33.3)	11 (47.8)	0.31
In-hospital mortality	21 (44.7)	11 (45.8)	10 (43.5)	0.87

APACHE = acute physiology and chronic health evaluation; CAP = community-acquired pneumonia; COPD = chronic obstructive lung disease; HAP = hospital-acquired pneumonia; HCAP = healthcare-associated pneumonia; ICU = intensive care unit; SOFA = sequential organ failure assessment.

Data are reported as n (%), otherwise stated.

a : Some patients had one or more underlying disease or condition.

### Pathogens

Pathogens that were identified in 47 patients are summarized in Table 2. Twenty-eight bacterial pathogens were identified in 24 patients. In 4 patients, two different bacteria were identified. *Staphylococcus aureus* (n = 5) was the most common bacteria, followed by *Legionella pneumophila* (n = 4), and *Streptococcus pneumoniae* (n = 3). Bacteria were identified from BAL fluid cultures or PCRs in 15 patients, from endotracheal aspirates or sputum cultures in 15 patients, from blood cultures in 4 patients, and from urinary antigen tests in 4 patients (two patients with pneumococcal antigens and two patients with legionella antigens). Eleven patients had two or more positive tests. Twenty-six viruses were identified in 23 patients. In three patients, two different viruses were identified. Rhinovirus was the most common virus (n = 11), followed by influenza virus (n = 6), and respiratory syncytial virus (n = 3). Viruses were identified from BAL fluid specimens in 18 patients and from nasopharyngeal specimens in 13 patients. Viruses were detected in both in BAL fluid and nasopharyngeal samples in 8 patients.

**Table 2:** Identities of pathogens in patients with pneumonia.

Group	Pathogen	Number
Bacterial pneumonia, (n = 24)^a^	*Staphylococcus aureus*	5
	*Legionella pneumophila*	4
	*Streptococcus pneumoniae*	3
	*Haemophilus influenzae*	1
	*Mycoplama pneumoniae*	1
	Enteric gram-negative bacilli	8
	* Escherichia coli*	4
	*Klebsiella pneumoniae*	3
	*Enterobacter cloacae*	1
	* Proteus mirabilis*	1
	* P. stuartii*	1
	Non-fermenting gram-negative bacilli	4
	* Acinetobacter baumannii*	3
	* Pseudomonas aeruginosa*	1
Viral pneumonia, (n = 23)^b^	Rhinovirus	11
	Influenza virus	6
	Influenza A	5
	Influenza B	1
	Respiratory syncytial virus	3
	Respiratory syncytial virus A	2
	* *Respiratory syncytial virus B	1
	Parainfluenza virus	2
	Type 3	1
	Type 2	1
	Human coronavirus OC43/HKU1	2
	Human metapneumovirus	2
	* *Bocavirus	1

Data are presented as the number (percentage) of patients.

a: Twenty-eight bacterial pathogens were identified in 24 patients. In four patients, two different bacteria were identified (*S. pneumoniae* + *H. influenzae*, *S. aureus* + *K. pneumoniae*, *E. coli* + *E. cloacae*, and *P. mirabilis* + *P. stuartii)*

b : Twenty-six viruses were identified in 23 patients. In three patients, two differrent viruses were identified (influenza virus A + rhinovirus, influenza virus A + respiratory syncytial virus B, and rhinovirus + human coronavirus OC43/HKU1).

### Cellular profiles of BAL fluid

**Figure 2:**
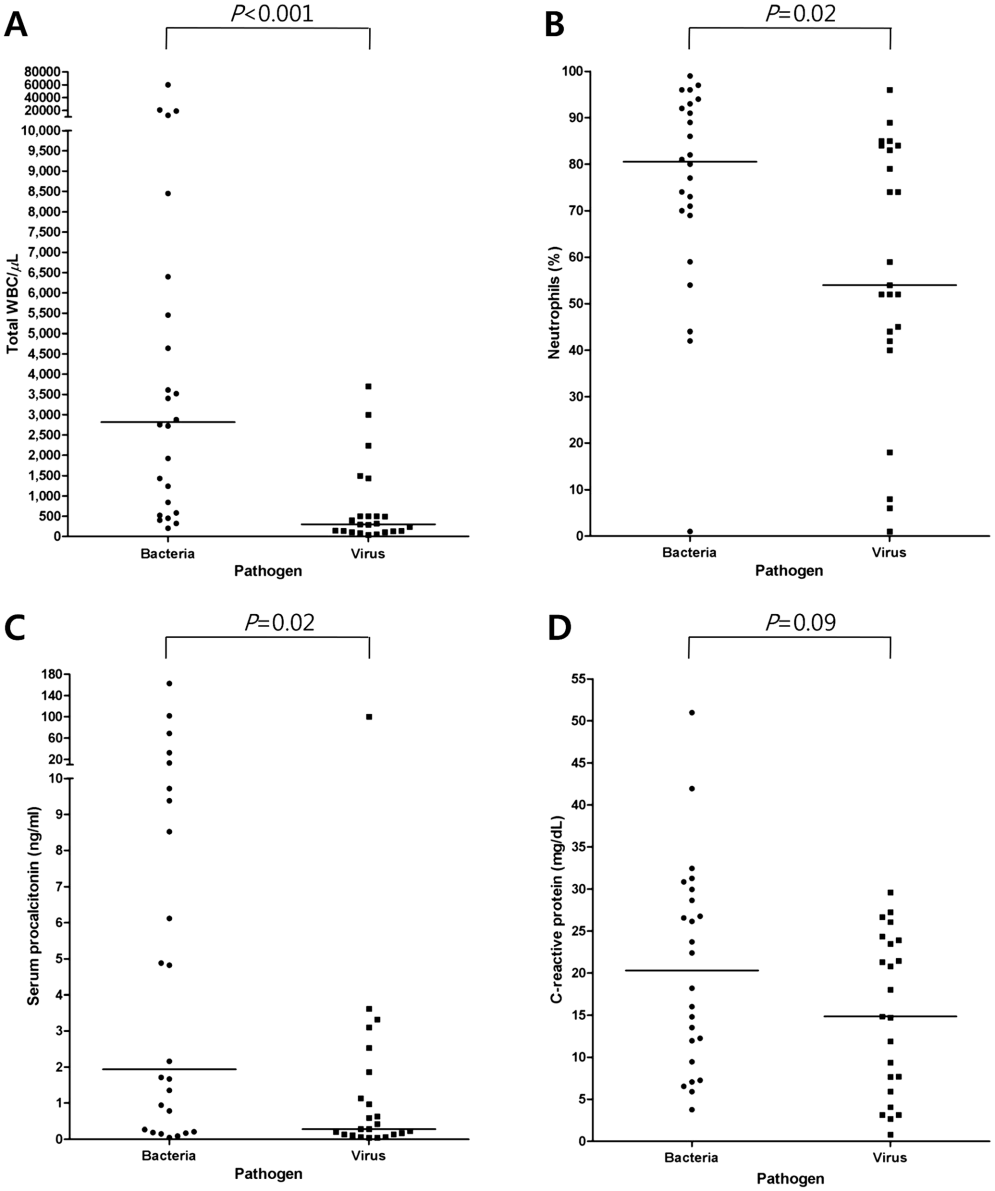
Distributions of bronchoalveolar lavage (BAL) fluid total white blood cell (WBC) count, BAL fluid percentage of neutrophils, serum procalcitonin concentration, and serum C-reactive protein concentration. **(A)** total WBC count in BAL fluid, **(B)** percentage of neutrophils in BAL fluid, **(C)** serum procalcitonin concentration, and **(D)** serum C-reactive protein concentration. Horizontal bars indicate median values.

Cellular BAL fluid profiles and distributions of BAL fluid cell counts in the two groups are shown in Table 3 and Figure 2. Detailed data of each patient are summarized in Table S1. The median total WBC count (2,815/µL vs. 300/µL, *P*<0.001), percentage of neutrophils (80.5% vs. 54.0%, *P* = 0.02), and absolute neutrophil count (2,661/µL vs. 204/µL, *P*<0.001) of BAL fluid were significantly higher in the bacterial pneumonia group than in the viral pneumonia group. The median serum procalcitonin concentration was also higher in the bacterial pneumonia group than in the viral pneumonia group (1.9 ng/ml vs. 0.3 ng/ml, *P* = 0.02), and the C-reactive protein concentration tended to be higher in the bacterial pneumonia group than in the viral pneumonia group (20.3 mg/dL vs. 14.9 mg/dL, *P* = 0.09).

**Table 3:** Bronchoalveolar lavage total and differential cell counts (%) in patients with pneumonia**.**

	Bacterial pneumonia (n = 24)	Viral pneumonia (n = 23)	*P*-value	Area under the ROC (95% confidence interval)
Total WBC count, cell/µL	2,815 (645–6,163)	300 (130–500)	<0.001	0.855 (0.750–0.960)
Neutrophils, %	80.5 (69.3–92.8)	54.0 (42.0–84.0)	0.02	0.701 (0.550–0.852)
Lymphocytes, %	4.0 (1.0–8.0)	8.0 (4.0–12.0)	0.02	0.305 (0.154–0.456)
Macrophages, %	12.0 (3.3–22.5)	20.0 (9.0–41.0)	0.04	0.324 (0.168–0.480)
Neutrophils count, cell/µL	2,661 (344–5636)	204 (48–480)	<0.001	0.837 (0.724–0.950)
Lymphocytes count, cell/µL	61 (17–274)	25 (11–58)	0.08	0.651 (0.487–0.816)
Macrophages count, cell/µL	144 (99–624)	62 (18–160)	0.004	0.743 (0.600–0.886)
Serum procalcitonin concentration, ng/ml	1.9 (0.2–9.6)	0.3 (0.1–1.9)	0.02	0.705 (0.554–0.855)
Serum C-reactive protein concentration, mg/dL	20.3 (10.1–29.6)	14.9 (5.9–23.9)	0.09	0.645 (0.487–0.803)

ROC  =  receiver operating characteristic curve. WBC  =  white blood cell.

Data are presented as median (interquartile range).

Of the 100 pathogen-identified patients who had received antimicrobial agent for more than 24 hours prior to bronchoscopic BAL (Figure 1), 44 had bacterial pneumonia, 54 had viral pneumonia, and 2 had invasive pulmonary aspergillosis. Of the 98 patients with bacterial pneumonia or viral pneumonia, the median duration of antimicrobial therapy before bronchoscopic BAL was 5 days (interquartile range, 3–9 days). The median total WBC count (395/µL vs. 200/µL, *P* = 0.52) and percentage of neutrophils (69.0% vs. 67.0%, *P* = 0.26) were not significantly different between these two groups. Figure S1 shows the changes recorded in the BAL fluid total WBC count and percentage of neutrophils according to the duration of exposure to antimicrobial agents.

### Diagnostic performances of BAL fluid cellular components, serum procalcitonin concentration, and serum C-reactive protein concentration for the prediction of bacterial pneumonia

**Figure 3:**
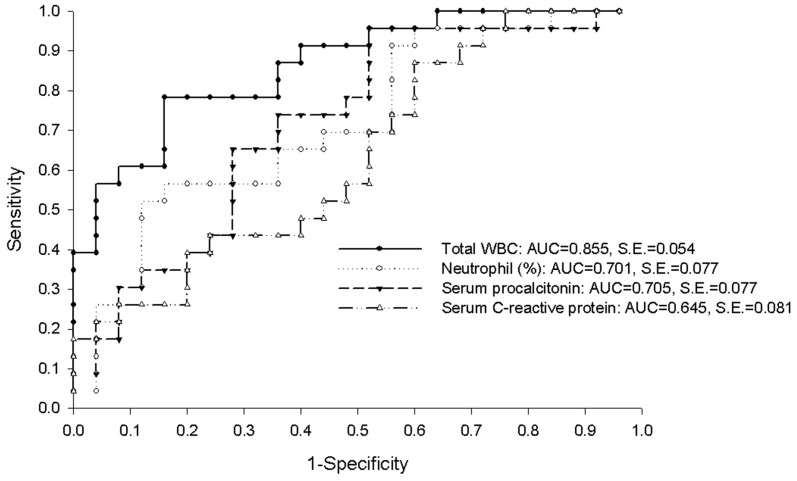
Receiver-operating curves of bronchoalveolar lavage fluid (BAL) total white blood cell count, BAL fluid percentage of neutrophils, serum procalcitonin concentration, and serum C-reactive protein concentration for differentiating between bacterial pneumonia and viral pneumonia.

The ability of BAL fluid cellular analysis to distinguish between bacterial pneumonia and viral pneumonia was assessed using ROC analysis (Table 3 last column and Figure 3). Total WBC count yielded the largest area under the ROC curve (AUC = 0.855, 95% confidence interval [CI] 0.750–0.960]; *P*<0.001), followed by neutrophil count (AUC = 0.837, 95% CI; 0.724–0.950, *P*<0.001), and percentage of neutrophils (AUC = 0.701, 95% CI; 0.550–0.852, *P* = 0.02). The diagnostic values of BAL fluid cellular components were better than those of serum procalcitonin concentration (AUC = 0.705, 95% CI; 0.554–0.855) and C-reactive protein concentration (AUC = 0.645, 95% CI; 0.487–0.803).

The sensitivities, specificities, positive predictive values, negative predictive values, positive likelihood ratios, and negative likelihood ratios are summarized in Table 4. When the cutoff value of BAL fluid total WBC count was ≥510/µL, which was selected using Youden’s Index, sensitivity was 83.3% (95% CI; 67.9–93.2), specificity was 78.3% (95% CI; 62.2–88.6), positive predictive value was 68.2% (95% CI; 49.2–82.6), negative predictive value was 89.3% (95% CI; 77.0–95.5), positive likelihood ratio was 3.83 (95% CI; 1.80–8.17), and negative likelihood ratio was 0.21 (95% CI; 0.08–0.52). A combination of BAL fluid total WBC count ≥510/µL or serum procalcitonin concentration ≥0.71 ng/mL had a sensitivity of 95.8% (95% CI; 81.6–99.8) and a negative likelihood ratio of 0.07 (95% CI; 0.003–0.40), whereas BAL fluid total WBC count ≥510/µL and serum C-reactive protein concentration ≥26.1 mg/dl had specificity of 95.7% (95% CI, 81.6–99.8) and a positive likelihood ratio of 8.63 (95% CI, 1.31–180.96).

**Table 4:** Accuracy of bacterial pneumonia diagnosis in patients with pneumonia.

	Sensitivity		Specificity					
Predictor	n/N	Percentile (95% CI)	n/N	Percentile (95% CI)	PPV (95% CI)^a^	NPV (95% CI)^a^	PLR (95% CI)	NLR (95% CI)
Total WBC count ≥510 cell/µL^b^	20/24	83.3% (67.9–93.2)	18/23	78.3% (62.2–88.6)	68.2% (49.2–82.6)	89.3% (77.0–95.5)	3.83 (1.80–8.17)	0.21 (0.08–0.52)
Neutrophils ≥64%^b^	19/24	79.2% (63.5–90.9)	13/23	56.5% (40.2–68.8)	50.5% (38.0–62.9)	82.9% (67.3–92.0)	1.82 (1.06–2.92)	0.37 (0.13–0.91)
Serum procalcitonin concentration ≥0.7 ng/ml^b^	17/24	70.8% (54.8–83.8)	15/23	65.2% (48.5–78.7)	53.3% (38.1–67.9)	80.0% (66.7–88.9)	2.04 (1.06–3.94)	0.45 (0.21–0.93)
Serum C-reactive protein concentration ≥26.1 mg/dl^b^	10/24	41.7% (27.1–50.7)	20/23	87.0% (71.7–96.3)	64.2% (36.0–85.1)	72.7% (64.7–79.5)	3.19 (0.96–13.83)	0.59 (0.49–0.65)
Total WBC count ≥510 cell/µL and serum procalcitonin concentration ≥0.71 ng/ml	14/24	58.3% (42.7–68.9)	19/23	82.6% (66.3–93.6)	65.3% (42.0–83.0)	78.0% (68.0–85.5)	3.35 (1.27–10.84)	0.50 (0.33–0.86)
Total WBC count ≥510 cell/µL or serum serum procalcitonin ≥0.71 ng/ml	23/24	95.8% (81.6–99.8)	14/23	60.9% (46.0–65.0)	57.8% (45.0–70.0)	96.3% (78.8–99.5)	2.45 (1.51–2.85)	0.07 (0.003–0.40)
Total WBC count ≥510 cell/µL and Serum C-reactive protein ≥26.1 mg/dl	9/24	37.5% (24.1–41.4)	22/23	95.7% (81.6–99.8)	82.9% (39.9–97.2)	73.2% (66.5–79.0)	8.63 (1.31–180.96)	0.65 (0.59–0.93)
Total WBC count ≥510 cell/µL or Serum C-reactive protein concentration ≥11.94 mg/dl	21/24	87.5% (72.2–96.4)	16/23	69.6% (53.6–78.8)	61.7% (46.0–75.3)	90.9% (76.9–96.7)	2.88 (1.56–4.55)	0.18 (0.05–0.52)

*Definition of abbreviations*: NLR  =  negative likelihood ratio; NPV  =  negative predictive value; PLR  =  positive likelihood ratio; PPV  =  positive predictive value; WBC  =  white blood cell.

a : Prevalence of bacterial pneumonia was assumed to be 35.9%, based on our previous study.

b : Area under the curve using cutoff point selected by Youden’s index (sensitivity+specificity-1).

When a cutoff value of BAL fluid total WBC count ≥510/µL was applied to 100 pathogen-identified patients who had received antimicrobial agent for more than 24 hours, sensitivity was 36.8% (95% CI; 21.8–54.0), specificity was 71.4% (95% CI; 56.7–83.4), positive predictive value was 41.9% (95% CI; 28.2–57.0), negative predictive value was 66.9% (95% CI; 59.9–73.2), positive likelihood ratio was 1.29 (95% CI; 0.70–2.37), and negative likelihood ratio was 0.89 (95% CI; 0.66–1.19).

**Figure 4:**
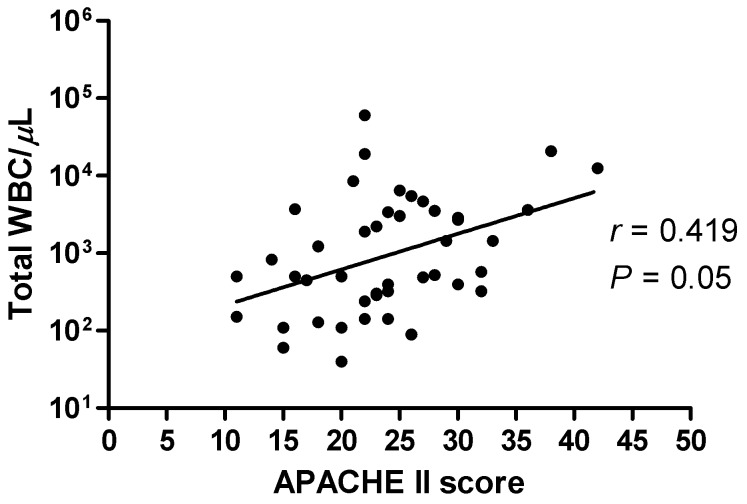
Correlation between bronchoalveolar lavage total white blood cell count and APACHE II score.

Multiple logistic regression analysis revealed that BAL fluid total WBC count ≥510/µL was an independent predictor of bacterial pneumonia with an adjusted odds ratio of 13.5 (95% CI; 2.3–80.4) (Table 5). There was a modest but significant positive correlation between the degree of BAL leukocytosis and the APACHE II score (*r* = 0.419, *P* = 0.05) (Figure 4).

**Table 5:** Multiple logistic-regression analysis of predictors for the diagnosis of bacterial pneumonia.

Predictor	Odds ratio	95% Confidence interval	*P*-value
Total WBC count ≥510 cell/µL	13.5	2.3–80.4	0.004
Serum procalcitonin concentration ≥0.71 ng/ml	2.6	0.4–17.7	0.32
Serum C-reactive protein ≥26.1 mg/dL	5.0	0.5–47.1	0.16
APACHE II score ≥24.5	5.4	0.7–42.2	0.11
Septic shock at admission	0.9	0.1–6.3	0.91

APACHE = acute physiology and chronic health evaluation;WBC = white blood cell.

## Discussion

This study analyzed the usefulness of cellular analysis of BAL fluid for predicting the etiology of pneumonia in critically ill adult patients. Neutrophilic pleocytosis in BAL fluid was frequently found in patients with bacterial- and viral pneumonia. The degree of pleocytosis, which was higher in the bacterial pneumonia, was useful for differential diagnosis of bacterial pneumonia. Total WBC count had the best diagnostic accuracy for predicting bacterial pneumonia, and its diagnostic performances was better than those of serum procalcitonin and C-reactive protein concentrations. Combinations of BAL fluid total WBC count, serum procalcitonin concentration, and serum C-reactive protein concentration provided the best diagnostic yields. The data suggest that cellular analysis of BAL fluid is a rapid and useful technique for differentiating bacterial pneumonia from viral pneumonia, and can be used to direct early appropriate treatment.

Information about the role of cellular profiles of BAL fluid for differential diagnosis of bacterial pneumonia in adult patients is limited. Stolz et al [11]. evaluated potential markers of bacterial infection in a cohort of immunocompromised patients with pulmonary complications. They reported that the percentage of neutrophils in BAL fluid and the serum procalcitonin concentration are independent predictors of bacterial infection. They suggested that the optimal cutoff value of the percentage of neutrophils in BAL fluid is 15% (sensitivity 84%; specificity 77%), which is much lower than the cutoff value in the current study. Sternberg et al.[13] investigated the usefulness of BAL in assessing pneumonia in renal transplant patients, and suggested that the optimal cutoff value of the percentage of neutrophils in BAL fluid is >20% for predicting bacterial pneumonia. However, neither of these previous studies included patients with severe pneumonia caused by respiratory viruses alone, and both compared BAL findings between patients with bacterial pneumonia and those with non-infectious diseases. In the current study, patients with viral pneumonia were included by using the newly developed multiplex respiratory virus RT-PCR. This showed that cases of viral pneumonia were frequently associated with neutrophilia in BAL fluid (median 54.0%). We speculate that this underlies why the optimal cutoff value of percentage of neutrophils in BAL fluid for predicting bacterial pneumonia is much higher in the current study (64%, Table 4) than in previous studies. Several authors of the current study previously investigated the diagnostic utility of soluble triggering receptor expressed on myeloid cells-1 (sTREM-1) in BAL fluid of various patient populations with bilateral lung infiltrates. A cutoff value of ≥60% neutrophils in BAL fluid is useful for differential diagnosis of bacterial or fungal pneumonia from other causes of pneumonia or non-infectious diseases (AUC = 0.77, 95% CI; 0.54–0.84, *P* = 0.001) [12]. In comparison to this previous study, the current study did not include patients with non-infectious diseases or fungal pneumonia, included much more cases of severe viral pneumonia, and analyzed the counts of various cell types.

Among the currently available inflammatory markers, serum procalcitonin is one of the best indicators of bacterial infections, including lower respiratory tract infections [19]. The usefulness of serum procalcitonin measurements has been validated in the diagnosis, severity assessment, and follow-up of patients with lower respiratory tract infections [20]–[22]. In the current study, the AUC of serum procalcitonin concentration for predicting bacterial pneumonia (AUC = 0.705) was smaller than those of total WBC (AUC = 0.855) and neutrophil (AUC = 0.837) counts. The combination of BAL fluid WBC counts and serum procalcitonin concentration tended to improve the diagnostic accuracy of the ROC model. This indicates that combinations of these markers can be useful to rule-out (BAL fluid total WBC count ≥510/µL or serum procalcitonin concentration ≥0.71 ng/mL with a sensitivity of 95.8% and negative likelihood ratio of 0.068) or rule-in (BAL fluid total WBC count ≥510/µL and serum C-reactive protein concentration ≥26.1 mg/dl with a specificity of 95.7% and positive likelihood ratio of 8.63) bacterial pneumonia. Diagnostic accuracy could be further improved if BAL fluid cellular profiles are interpreted alongside clinical presentations, radiographic studies, and other relevant test results. Using this approach, it might be possible to identify patients who can be managed without antibacterial agents or those who require antiviral agents.

Although not included in the current study, sTREM-1 in BAL fluid is another notable biomarker for the diagnosis of pneumonia. sTREM-1 is reportedly a potent discriminator of bacterial pneumonia from non-infectious lung infiltrations [12], [23]–[27]. However, the proposed cutoff values of sTREM-1 concentration vary widely (5–900 pg/ml) and some studies have questioned the reliability of BAL fluid sTREM-1 [28]–[30]. Studies on BAL fluid sTREM-1 have mainly been confined to patients with ventilator-associated pneumonia. Therefore, the usefulness of sTREM-1 for etiologic diagnosis of pneumonia, especially differential diagnosis of viral pneumonia, has not been elucidated yet. The current study directly compared patients with bacterial and viral pneumonia, and therefore differs from previous studies of sTREM-1. BAL fluid sTREM-1 concentration in combination with the BAL fluid cellular profile might exhibit better diagnostic performance, although this warrants further studies.

A strength of the current study is the relatively strict enrollment criteria used. To minimize bias associated with antimicrobial therapy, all patients who received antimicrobial therapy for more than 24 hours were excluded, regardless of the adequacy of prior antimicrobial therapy. By using strict enrollment criteria, however, only a small proportion of pneumonia patients who underwent bronchoscopic BAL was finally included (Figure 1), which might have influenced on the results. However, positive likelihood and negative likelihood ratio, which are not influenced by disease prevalence, were good, which supports the validity of the data. In clinical practice, the results might be applied to patients who have received antimicrobial therapy more than 24 hours. That is, if BAL fluid cellular analysis shows evident pleocytosis even after antimicrobial therapy for more than 24 hours, it would be a strong suggestion for bacterial etiology.

The study has several limitations. First, the small sample size of the select critically ill patient population analyzed limits the general applicability of our findings. Moreover, since our study included critically ill patients with acute respiratory failure secondary to pneumonia who were not receiving antimicrobial therapy, our results may not be applicable to the majority of severe pneumonia patients in clinical practice. Second, the impact of antimicrobial therapy on the both BAL fluid cellular profiles and other inflammation markers such as procalcitonin, remains to be further elucidated. Third, cases of invasive pulmonary aspergillosis, *Pneumocystis jirovecii* pneumonia, and mycobacterial pneumonia, were not included, mainly because few patients had these types of pneumonia. Fourth, patients with non-infectious causes of pulmonary infiltrates that can often mimic infectious causes, such as acute respiratory distress syndrome, cryptogenic organizing pneumonia, eosinophilic pneumonia, and drug-induced pneumonitis, were also excluded from our analyses. The inclusion of those cases may have caused a marked decrease in the specificity of our BAL fluid criteria. Finally, all the pathogens were not directly identified from BAL fluid. Some patients were included in whom pathogens were identified by other means, such as blood culture, endotracheal aspirates culture, urinary pneumococcal antigen test, and PCR from nasopharyngeal samples, as long as clinically and radiographically compatible and no other etiology was demonstrated. Therefore, patients with coincidental upper respiratory infections or colonization may have been included.

In conclusion, the data indicate that cellular analysis of BAL fluid, alone or in combination with serum procalcitonin and C-reactive protein concentrations, may rapidly provide valuable diagnostic information for the early differential diagnosis of pneumonia in critically ill adult patients.

## Supporting Information

Figure S1 Changes in the bronchoalveolar lavage fluid (A) total white blood cell (WBC) count and (B) percentage of neutrophils according to the duration of exposure to antimicrobial agents (median plus interquartile range).
(TIF)

Table S1 Characteristics and cellular profiles of bronchoalveolar lavage fluid in patients with pneumonia.
(DOCX)
