# Supplementary figures and images for: Usefulness of Cellular Analysis of Bronchoalveolar Lavage Fluid for Predicting the Etiology of Pneumonia in Critically Ill Patients
Source: PLoS One. 2014 May 13;9(5):e97346. doi: 10.1371/journal.pone.0097346 (PMC4019586; doi:10.1371/journal.pone.0097346)

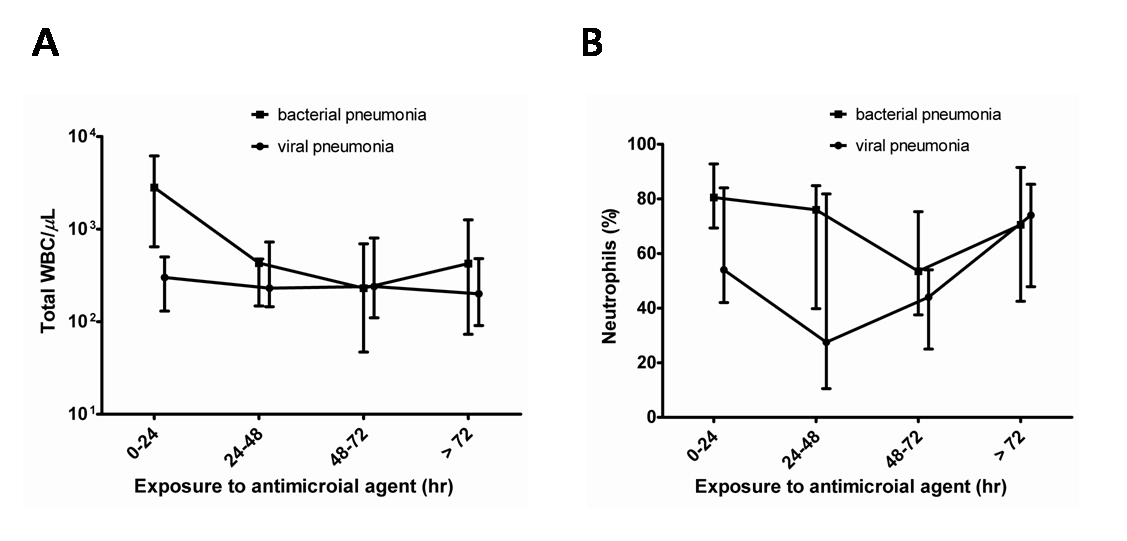

Supplement: Figure S1 — Changes in the bronchoalveolar lavage fluid (A) total white blood cell (WBC) count and (B) percentage of neutrophils according to the duration of exposure to antimicrobial agents (median plus interquartile range). (TIF) [file pone.0097346.s001.tif]
