# Supplementary material for: Usefulness of Cellular Analysis of Bronchoalveolar Lavage Fluid for Predicting the Etiology of Pneumonia in Critically Ill Patients
Source: PLoS One. 2014 May 13;9(5):e97346. doi: 10.1371/journal.pone.0097346 (PMC4019586; doi:10.1371/journal.pone.0097346)
Supplement: Table S1 — Characteristics and cellular profiles of bronchoalveolar lavage fluid in patients with pneumonia. (DOCX) [file pone.0097346.s002.docx]

**Table S1.** Characteristics and cellular profiles of bronchoalveolar lavage fluid in patients with pneumonia.

|  |  |  |  |  | **BAL fluid profile** | | | | | | |
| --- | --- | --- | --- | --- | --- | --- | --- | --- | --- | --- | --- |
| **Number** | **Sex/age** | **Remarkable underlying illness** | **Type of pneumonia** | **Pathogen(s)** | **RBC count, cell/µL** | **Total WBC count, cell/µL** | **Neutrophils, %** | **Lymphocytes, %** | **Macrophages, %** | **Serum procalcitonin concentration, ng/ml** | **C-reactive protein concentration, mg/dL** |
| Bacterial pneumonia (n=24) | | |  |  |  |  |  |  |  |  |  |
| 1 | M/55 | Lung cancer | HCAP | *S. aureus* | 9,920 | 1,920 | 42 | 4 | 54 | 8.52 | 28.66 |
| 2 | F/72 | Bronchial asthma | HAP | *S. aureus* | 0 | 400 | 71 | 0 | 29 | 0.94 | 5.89 |
| 3 | F/75 | Interstitial lung disease | HAP | *S. aureus* | 1,160 | 1,240 | 69 | 4 | 27 | 0.21 | 14.81 |
| 4 | M/75 | Interstitial lung disease | HCAP | *S. aureus* | 54,000 | 520 | 54 | 13 | 29 | 0.17 | 9.46 |
| 5 | M/57 | Burn | HAP | *S. aureus* + *K. pneumoniae* | 21,500 | 840 | 59 | 5 | 19 | 0.27 | 30.87 |
| 6 | F/68 | Rheumatoid arthritis | HCAP | *Legionella* species | 650 | 2,750 | 1 | 93 | 5 | 0.09 | 16.02 |
| 7 | M/70 | Astrocytoma | HCAP | *Legionella* species | 480,000 | 19,200 | 91 | 0 | 7 | 1.71 | 29.97 |
| 8 | M/67 | s/p kidney transplantation | HAP | *Legionella* species | 9,000 | 2,720 | 94 | 5 | 1 | 162.64 | 26.57 |
| 9 | M/77 | Lung cancer | HCAP | *Legionella* species | 2,400 | 3,520 | 89 | 3 | 6 | 4.88 | 41.95 |
| 10 | M/42 | None | CAP | *S. pneumoniae* | 60,000 | 2,880 | 96 | 0 | 4 | 101.77 | 50.99 |
| 11 | M/83 | Idiopathic thrombocytopenic purpura | CAP | *S. pneumoniae* | 16,960 | 20,800 | 73 | 4 | 23 | 13.38 | 32.48 |
| 12 | M/75 | Alcoholism | CAP | *S. pneumoniae* + *H. influenzae* | 320 | 3,610 | 97 | 0 | 3 | 9.72 | 26.76 |
| 13 | M/22 | None | CAP | *M. pneumoniae* | 1,100 | 450 | 70 | 8 | 21 | 2.16 | 31.29 |
| 14 | M/60 | Liver cirrhosis | CAP | *K. pneumoniae* | 23,040 | 320 | 77 | 6 | 17 | 4.82 | 12.26 |
| 15 | M/67 | Bronchiectasis | HCAP | *K. pneumoniae* | 1,280 | 6,400 | 93 | 5 | 2 | 6.12 | 11.97 |
| 16 | M/72 | COPD | CAP | *E. coli* | 2,800 | 5,450 | 86 | 1 | 13 | 32.53 | 7.25 |
| 17 | M/51 | Colon cancer | CAP | *E. coli* | 1,340 | 1,430 | 80 | 8 | 11 | 68.87 | 22.40 |
| 18 | F/77 | Diabetes mellitus, ESRD | HCAP | *E. coli* | 43,520 | 4,640 | 82 | 10 | 8 | 1.67 | 23.72 |
| 19 | M/52 | Esophageal cancer | HCAP | *E. coli* + *E. cloacae* | 100 | 60,000 | 96 | 1 | 3 | 0.15 | 18.20 |
| 20 | M/62 | COPD | HCAP | *P. mirabilis* + P. *stuartii* | 48,000 | 3,400 | 92 | 4 | 3 | 1.35 | 26.16 |
| 21 | M/59 | Intestinal amyloidosis | HAP | *A. baumannii* | 7,500 | 580 | 74 | 9 | 17 | 9.38 | 13.54 |
| 22 | M/22 | Aplastic anemia, s/p stem cell transplantation | HCAP | *A. baumannii* | 1,300 | 8,450 | 99 | 0 | 1 | 0.78 | 6.52 |
| 23 | F/69 | Interstitial lung disease | HCAP | *A. baumannii* | 1,520 | 200 | 44 | 8 | 42 | 0.19 | 7.06 |
| 24 | F/74 | COPD | HCAP | *P. aeruginosa* | 500 | 12,500 | 81 | 3 | 16 | 0.05 | 3.75 |
|  | | |  |  |  |  |  |  |  |  |  |
| Viral pneumonia (n=23) | | |  |  |  |  |  |  |  |  |  |
| 25 | M/73 | COPD | CAP | Rhinovirus | 1,520 | 500 | 84 | 10 | 3 | 1.86 | 24.37 |
| 26 | M/70 | Interstitial lung disease | HCAP | Rhinovirus | 800 | 240 | 85 | 6 | 9 | 0.17 | 2.65 |
| 27 | M/69 | None | CAP | Rhinovirus | 10 | 150 | 6 | 70 | 20 | 0.23 | 26.09 |
| 28 | M/51 | AML, s/p stem cell transplantation | HCAP | Rhinovirus | 14 | 140 | 40 | 12 | 44 | 0.05 | 7.68 |
| 29 | F/53 | AML, s/p stem cell transplantation | HCAP | Rhinovirus | 5,200 | 3.000 | 79 | 4 | 17 | 0.21 | 14.72 |
| 30 | M/62 | Interstitial lung disease | CAP | Rhinovirus | 800 | 60 | 52 | 18 | 30 | 0.28 | 29.63 |
| 31 | M/49 | Rheumatoid arthritis | HCAP | Rhinovirus | 40 | 500 | 83 | 5 | 7 | 0.28 | 18.03 |
| 32 | F/66 | Diabetes mellitus, rheumatoid arthritis | CAP | Rhinovirus | 1,550 | 90 | 74 | 11 | 15 | 3.62 | 23.49 |
| 33 | F/70 | Rheumatoid arthritis | HCAP | Rhinovirus | 5,200 | 400 | 52 | 10 | 38 | 0.42 | 9.36 |
| 34 | F/61 | Lung cancer | HCAP | Rhinovirus + Influenza A (2009 pdm H1N1) | 3,050 | 300 | 18 | 33 | 36 | 0.06 | 5.91 |
| 35 | M/62 | Diabetest mellitus | CAP | Influenza A (H3N2) | 1,760 | 2,240 | 85 | 2 | 13 | 100.10 | 26.67 |
| 36 | F/49 | Multiple myeloma | HAP | Influenza A (2009 pdm H1N1) | 10,000 | 500 | 96 | 2 | 2 | 1.13 | 20.83 |
| 37 | F/17 | AML, s/p stem cell transplantation | HCAP | Influenza A (2009 pdm H1N1) | 270 | 140 | 8 | 8 | 84 | 0.63 | 0.82 |
| 38 | F/82 | Interstitial lung disease | CAP | Influenza B | 11,520 | 40 | 59 | 4 | 37 | 0.11 | 21.32 |
| 39 | F/53 | Interstitial lung disease | HCAP | Influenza A (untyped) + respiratory syncytial virus B | 230 | 130 | 52 | 6 | 41 | 0.97 | 4.06 |
| 40 | M/80 | Diabetes mellitus | CAP | Respiratory syncytial virus A | 2,240 | 320 | 84 | 12 | 0 | 0.14 | 3.12 |
| 41 | M/67 | s/p kidney transplantation | HCAP | Respiratory syncytial virus A | 6,500 | 1,500 | 74 | 4 | 13 | 3.32 | 11.90 |
| 42 | M/35 | Hodgkin’s lymphoma | HCAP | Parainfluenza virus, type 3 | 1,380 | 110 | 44 | 22 | 18 | 0.14 | 14.85 |
| 43 | M/61 | Interstitial lung disease | CAP | Parainfluenza virus, type 2 | 23,000 | 110 | 1 | 24 | 72 | 0.06 | 7.66 |
| 44 | F/75 | Interstitial lung disease | HCAP | Human coronavirus-OC43/HKU1 | 5,060 | 490 | 42 | 5 | 53 | 0.05 | 3.14 |
| 45 | M/69 | Diabetes mellitus, s/p kidney transplantation | HCAP | Human coronavirus-OC43/HKU1 + Rhinovirus | 110,000 | 1,440 | 89 | 4 | 7 | 2.53 | 21.48 |
| 46 | M/72 | Insterstitial lung disease | HCAP | Human metapneumovirus | 80 | 3,700 | 54 | 8 | 35 | 3.10 | 23.91 |
| 47 | M/70 | Diabetes mellitus | CAP | Human metapneumovirus | 1,360 | 290 | 45 | 0 | 55 | 0.59 | 27.28 |

BAL=bronchoalveolar lavage; RBC=red blood cell; WBC=white blood cell; HCAP=healthcare-associated pneumonia; HAP=hospital-acquired pneumonia; CAP=community-acquired pneumonia; COPD=chronic obstructive lung disease; ESRD=end-stage renal disease; AML=acute myeloid leukemia.
